# 58096 A community-academic partnership to implement DASH diet and social/behavioral interventions in congregate meal settings to reduce hypertension among seniors aging in place

**DOI:** 10.1017/cts.2021.598

**Published:** 2021-03-30

**Authors:** Kimberly S. Vasquez, Adam Qureshi, Andrea Ronning, Moufdi Naji, Cameron Coffran, Clewert Sylvester, Glenis George-Alexander, Dacia Vasquez, Teeto Ezeonu, Chamanara Khalida, Victor Baez, William Dionne, Sharon Tobias, Debra Diaz, Caroline S. Jiang, Roger Vaughan, Barry S. Coller, Jonathan N. Tobin, Dozene Guishard, Rhonda G. Kost

**Affiliations:** 1 The Rockefeller University Center for Clinical and Translational Science; 2 Carter Burden Network; 3 Clinical Directors Network

## Abstract

ABSTRACT IMPACT: Our implementation model translates two evidence-based nutritional and behavioral interventions to lower blood pressure, into a community-based intervention program for seniors receiving congregate meals. OBJECTIVES/GOALS: The Rockefeller University, Clinical Directors Network, and Carter Burden Network received an Administration for Community Living Nutrition Innovation grant to test whether implementation of DASH-concordant meals and health education programs together lower blood pressure among seniors aging in place. METHODS/STUDY POPULATION: n=200, >60 yr, >4 meals/week at CBN; engagement of seniors/stakeholders in planning and conduct; Advisory Committee to facilitate dissemination; menus aligned with Dietary Approaches to Stop Hypertension (DASH) and NYC Department for the Aging nutritional guidelines; interactive sessions for education in nutrition, BP management, medication adherence. Training in use of automated daily home BP monitors (Omron 20). Validated surveys at M0, M1, M3, M6. Taste preference and cost assessed through Meal Satisfaction (Likert scale) and Plate Waste measures. Primary Outcome: Change in Systolic BP (SBP) at Month 1; change in %BP controlled. Secondary: validated cognitive, behavioral, nutritional measures (SF-12, PQH-2), economics; staff/client satisfaction, trends and significant associations. RESULTS/ANTICIPATED RESULTS: n=94, x^2^ age =73 +/- 8 years, 65% female, 50% White, 32% Black/African American, 4% Asian, 1% American Indian, Alaskan Native, 13% Other, 32% Latino/a, 43% with income <$20,000. Mean SBP at Baseline was 137.87 +18.8 mmHg (range 98-191). Menus were adapted to provide 20% daily DASH requirements at breakfast, 50% at lunch. Participants attended classes in nutrition and medication management and were provided with and trained to use an automated home BP monitor. Meal satisfaction scores dipped briefly then met or exceed pre-DASH levels. Home BP data was downloaded every 2-4 weeks with social/behavioral support. The COVID-19 closures interfered with BP outcome data collection and meal service ceased. Primary outcome: x^2^ change in SBP at Month 1 = -4.41 mmHg + 18 (n=61) (p=0.713). Significant associations will be reported. DISCUSSION/SIGNIFICANCE OF FINDINGS: Our community-academic research partnership implemented the DASH diet in congregate-meal settings to address uncontrolled hypertension in seniors. COVID-19 interrupted the study, but encouraging trends were observed that may inform refinement to this community-based health intervention for seniors.

